# Arthroscopic Lateral Ulnar Collateral Ligament Plication/Reconstruction With Augmented Lateral Collateral Ligament Imbrication

**DOI:** 10.1016/j.eats.2025.103529

**Published:** 2025-04-02

**Authors:** Christos Koukos, Dominique Schoeps, Joachim Windolf, In-Ho Jeon, Cristina Zolog, Konstantinos Ditsios, David Latz

**Affiliations:** a Sports Trauma and Pain Institute, Thessaloniki, Greece; b Department of Trauma Surgery and Orthopaedics, University Hospital Düsseldorf, Düsseldorf, Germany; c University Hospital Aachen, Aachen, Germany; d Department of Orthopaedic Surgery, Asan Medical Center, University of Ulsan, Seoul, Republic of Korea; e Department of Special Sports Surgery, Wuppertal, Germany; f 2nd Orthopaedic Department of Aristotle, University of Thessaloniki, "G Gennimatas" Hospital, Thessaloniki, Greece

## Abstract

Posterolateral rotatory instability (PLRI) is a chronic condition resulting from damage to the lateral ulnar collateral ligament (LUCL) and related structures causing painful restrictions and instability in elbow movement. Conservative treatment often falls short, necessitating surgical intervention to restore elbow stability. Traditionally, open LUCL reconstruction with tendon grafting has been the standard of care but involves substantial tissue disruption and extended recovery. Arthroscopic approaches offer a less-invasive alternative with reduced soft-tissue damage and faster recovery. This Technical Note presents an algorithm-driven, arthroscopically assisted technique for PLRI treatment. Using magnetic resonance imaging to evaluate the common extensor origin’s integrity, surgical options include LUCL plication with anchor fixation and lateral collateral ligament imbrication. During surgery, a minimally invasive technique is employed leveraging arthroscopy for precise ligament inspection and anchor-based fixation. Sutures stabilize the LUCL and lateral collateral ligament, addressing instability while preserving tissue integrity. This approach enables rapid postoperative rehabilitation and reduces the risk of motion restrictions. Although suitable for grade 1 and 2 PLRI, the technique demands high arthroscopic proficiency. This minimally invasive method allows effective management of elbow instability while promoting quicker patient recovery and long-term functional restoration.

The ulnohumeral joint is the primary stabilizer of the elbow; it is supported by the medial collateral ligament and the lateral collateral ligament (LCL) complex, including the annular ligament, lateral ulnar collateral ligament (LUCL), and radial collateral ligament.^1^ Posterolateral rotational instability (PLRI) results from LUCL damage, leading to painful restricted movement and chronic instability.^2^^,^^3^

PLRI typically occurs from trauma, falls, or complications after elbow surgery.^4^^,^^5^ Arthroscopic procedures and steroid injections can further weaken ligaments, increasing the risk of PLRI. Repetitive overuse injuries also may contribute, and untreated cases can lead to persistent instability in up to 20% of elbow injuries.^6^

Surgical intervention is necessary when conservative treatments like physiotherapy fail. Indications include recurrent dislocations, chronic pain, and functional impairment.^7^ Surgery reconstructs damaged structures to restore stability, often with the use of tendon grafts. Open LUCL reconstruction traditionally was favored but had longer recovery times and more tissue damage. Arthroscopic techniques have gained popularity as the result of reduced invasiveness and faster recovery.

Smith et al.^8^ introduced arthroscopic plication in 2001, using sutures near the lateral epicondyle. van Riet^9^ later modified this with double sutures for enhanced stabilization of the LCL, lateral capsule, and anconeus muscle.

Minimally invasive reconstruction with autologous or allogenic tendon grafts is preferred if the common extensor origin (CEO) is intact. More extensive procedures are required when the CEO is compromised to restore the extensor muscle origin. This Technical Note highlights an arthroscopically assisted approach for treating PLRI, providing a minimally invasive yet effective solution for this challenging condition.

## Surgical Technique

For the treatment of PRLI, an algorithm is used that considers the integrity of the CEO in the decision-making process.^10^ Initially, magnetic resonance imaging is performed preoperatively to assess the LUCL and CEO. Depending on the findings, the surgical procedure is chosen: If the CEO is intact or has a moderate partial tear, an arthroscopic LCL imbrication^11^ is performed. In cases of significant partial tear or a complete rupture without fiber retraction, an LCL plication with an anchor is carried out. If there is a complete rupture with fiber retraction, LUCL reconstruction with a triceps tendon graft is selected.^10^ We present the technique in the second stage of the algorithm, that is, arthroscopic LUCL plication with an anchor augmented with LCL imbrication.

**Fig 1:**
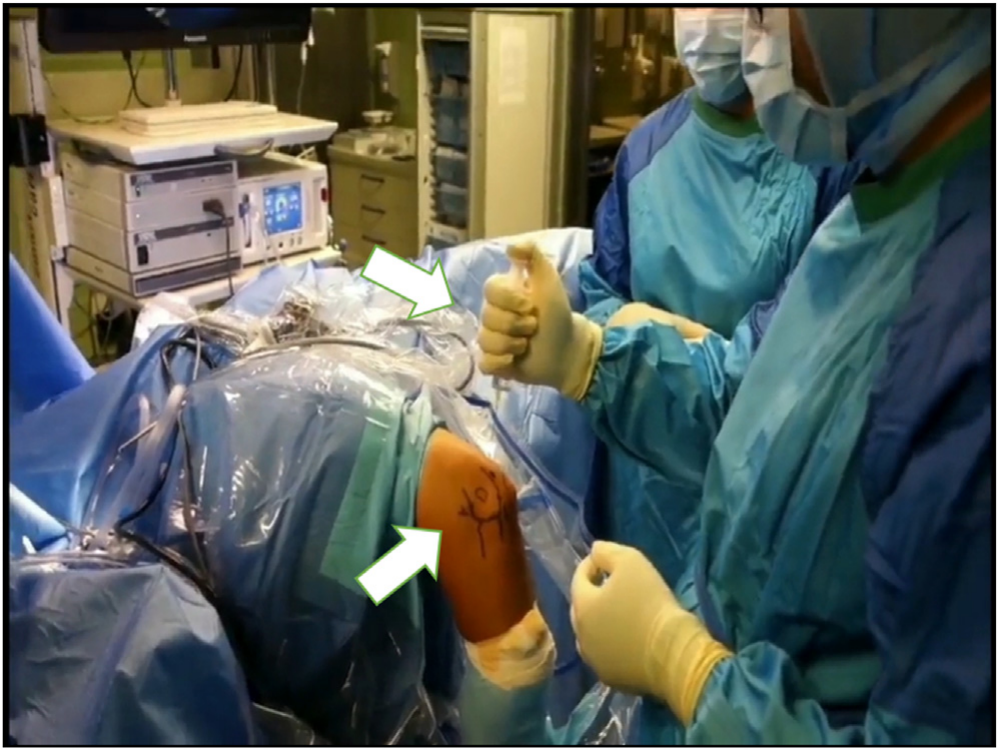
The patient is placed under general anesthesia and in a prone position (right side). An upper-arm pneumatic device is applied and the affected arm is fixed in an arm-resting device so that the forearm hangs down and the 90° flexed elbow is freely accessible. We can see that the important landmarks have been marked in advance and that the examiner is in the process of performing saline insufflation. Take-home message: proper positioning and preparation for the procedure are essential for a successful operation.

**Fig 2:**
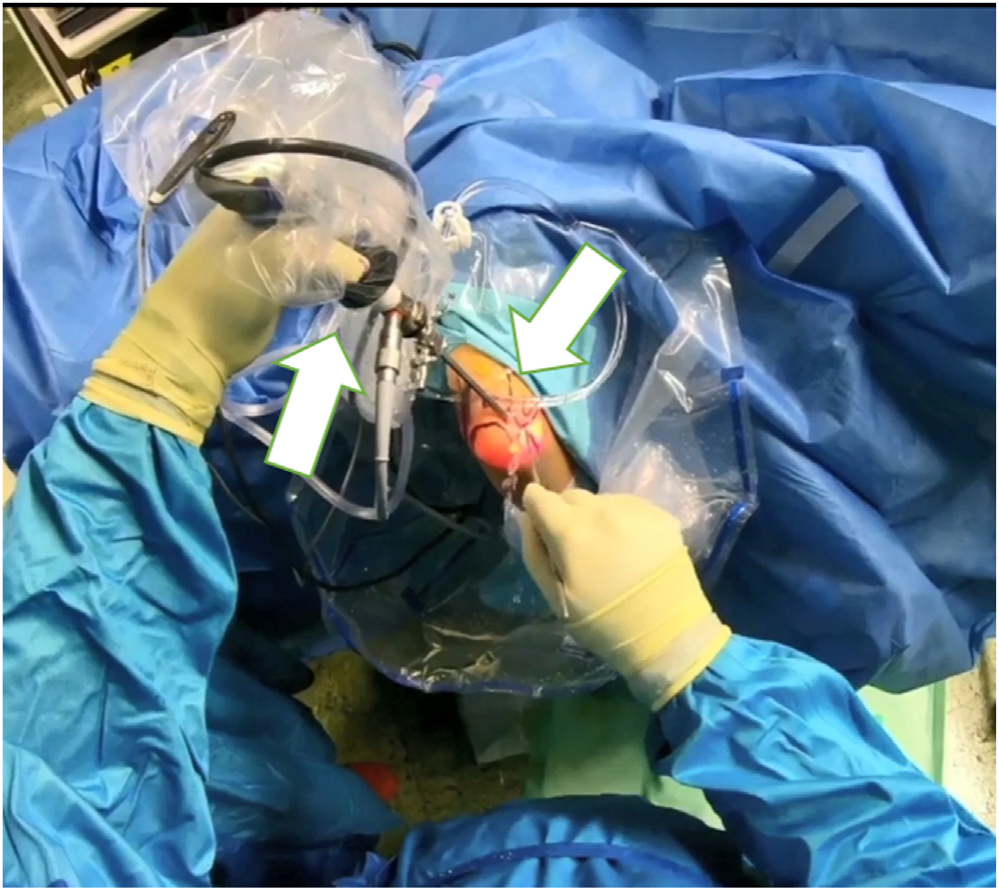
After the landmarks have been placed and the preoperative examination of the elbow has been performed under anesthesia, the elbow is now accessed arthroscopically via the posterolateral portal. Shown is the trocar in the left hand; an additional working portal is created with the right hand. Take-home message: After a clinical examination, standardized arthroscopic access to the elbow joint is performed first

The surgery is performed with the patient under general anesthesia and in the lateral decubitus position.^12^ After a preoperative examination (pivot-shift test, drawer test) and marking of key anatomical landmarks, the arthroscopic approach begins. An upper arm pneumatic device is applied and the affected arm is fixed in an arm-resting device so that the forearm hangs down and the 90° flexed elbow is freely accessible.^12^ The preoperative assessment includes documenting the range of motion and collateral ligament instability. Key elbow landmarks and the ulnar nerve course are marked before surgery (Fig 1). The tourniquet is then applied and access is prepared. After saline insufflation, the procedure begins with a posterior compartment view (Fig 1). A high posterolateral approach lateral to the triceps allows insertion of the trocar (Fig 2). From this perspective, the medial lateral ligament complex can be inspected under valgus stress. If intact, a medial approach is unnecessary.

Medial stability is tested via a transtricipital portal by inserting a 4-mm trocar between the medial olecranon tip and the trochlea. If it remains hinged without a medial opening, the medial collateral ligament is intact. The olecranon tip and fossa are visualized, and any pathology is treated through the central posterior portal.^8^ The scope is then placed in the radiohumeral gutter, and a soft spot portal is created for working access. Local debridement and synovectomy can be performed. Various arthroscopic tests assess rotational instability, confirming the PRLI.

**Fig 3:**
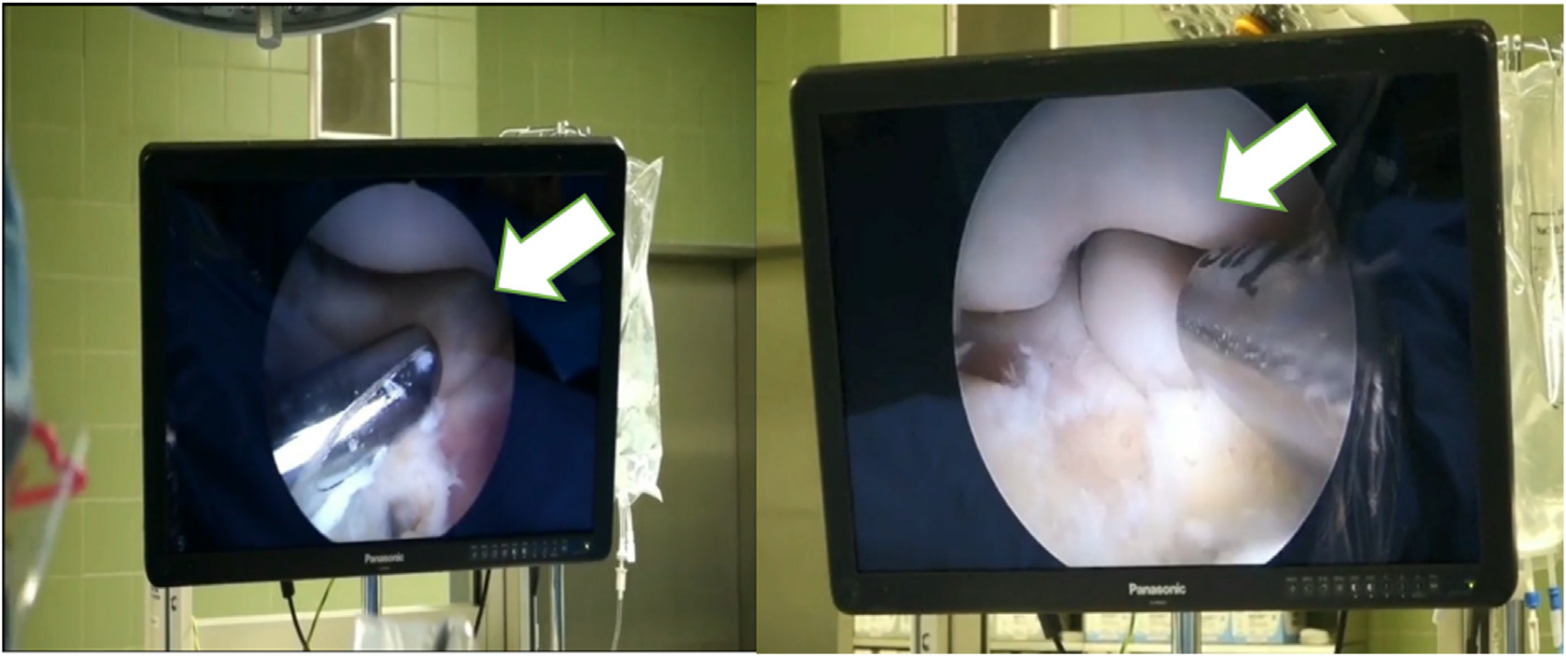
The camera's view during the arthroscopy. Once the working portal has been created, an overview of the situation can be obtained: the cartilage, synovia and ligaments can be examined and assessed. If necessary, this portal can be used for debridement and synovectomy. Take-home message: Pathologic abnormalities during the diagnostic round should be addressed by using the working portal for debridement.

Subsequently, the radiohumeral joint is accessed through a working portal to perform debridement and synovectomy (Fig 3, Video 1). Arthroscopic tests like the arthroscopic rotatory instability test^11^ are conducted to assess rotational instability. During excessive supination and varus stress, subluxation of the radial head can be observed, confirming instability. A loose annular ligament (loose collar sign) also may indicate lateral laxity.^8^ For significant partial or complete ruptures of the CEO, LUCL plication, with or without LCL imbrication, is performed to restore stability and improve elbow joint function.

### Arthroscopic Technique

**Fig 4:**
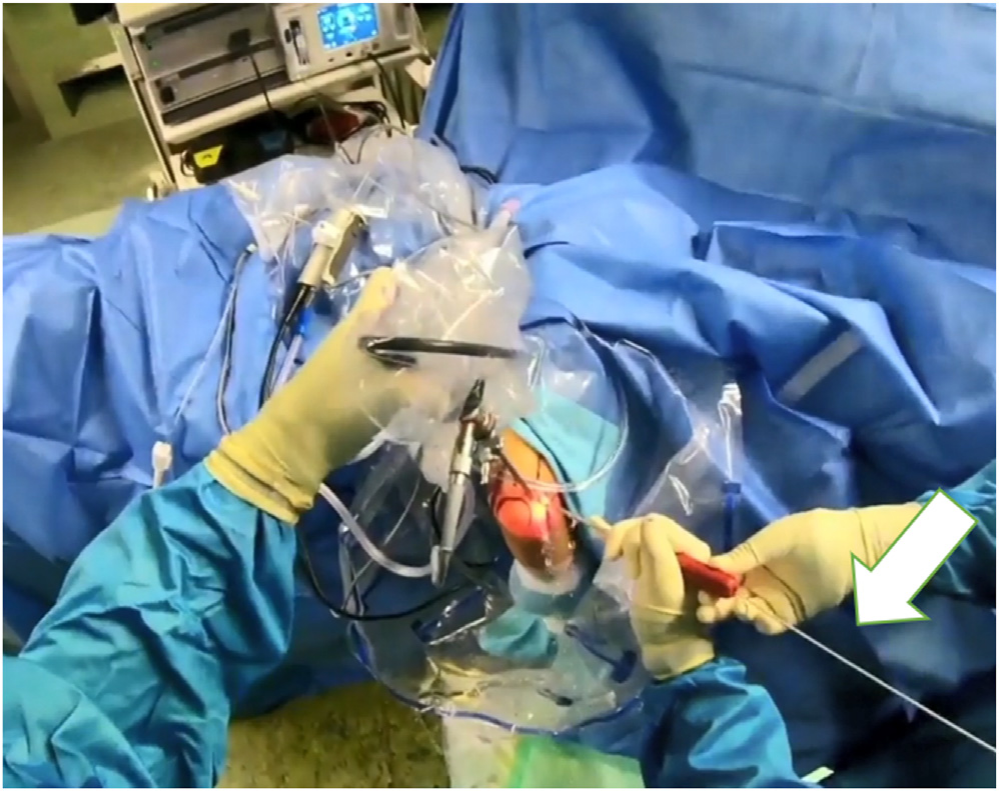
After identifying the injury origin, an anchor (1.8 FiberTak; Arthrex,) is inserted posterior to the isometric point of the lateral capitellum, behind the lateral ulnar collateral ligament origin.

**Fig 5:**
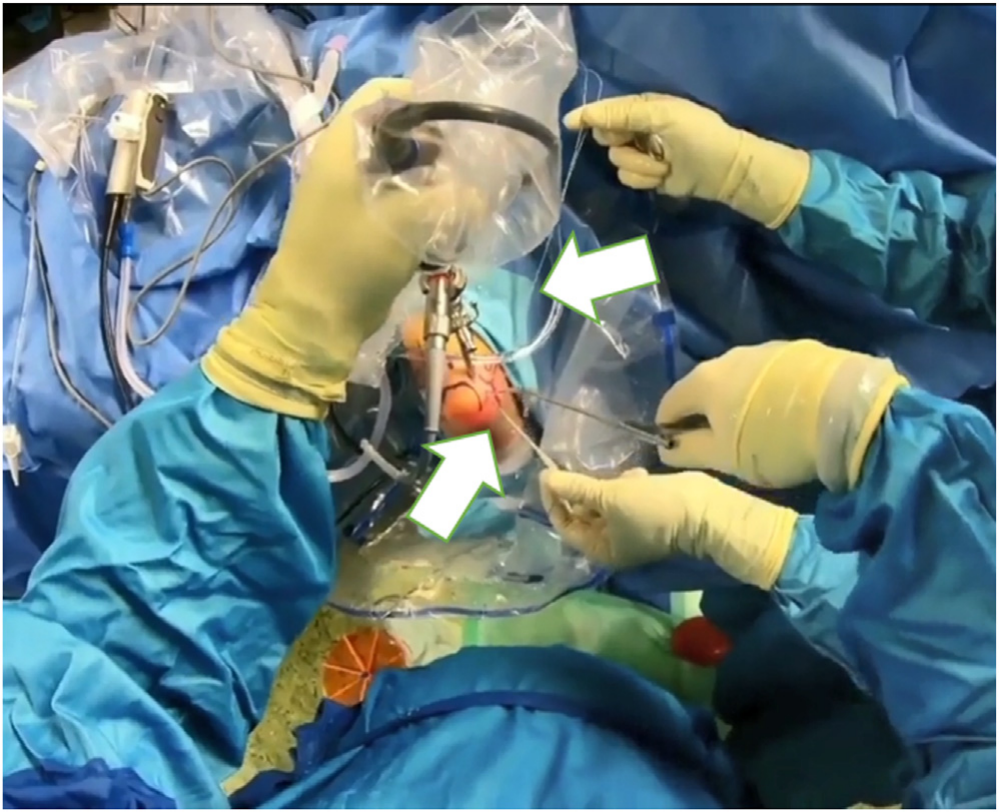
Sutures are passed through the radial collateral ligament, common extensor origin, and lateral ulnar collateral ligament using a 14-gauge needle as a suture passer, then retrieved subcutaneously through the accessory posterolateral portal

The LUCL attaches at the posterior corner of the lateral epicondyle, which should be carefully debrided using a motorized shaver (Fig 3, Video 1). After identifying the injury origin, an anchor (Fadenanker/kleine Gelenke, 1.8 FiberTak; Arthrex GmbH) is inserted posterior to the isometric point of the lateral capitellum, behind the LUCL origin (Fig 4, Video 1). Sutures are passed through the radial collateral ligament, CEO, and LUCL using a 14-gauge needle as a suture passer, then retrieved subcutaneously through the accessory posterolateral portal (Fig 5). The individual suture ends are placed so that horizontal mattress sutures pull toward the healthy part of the ligament. The sutures are kept under tension while the lateral gutter is inspected with the arthroscope. If residual instability persists, additional imbrication can be performed.

**Fig 6:**
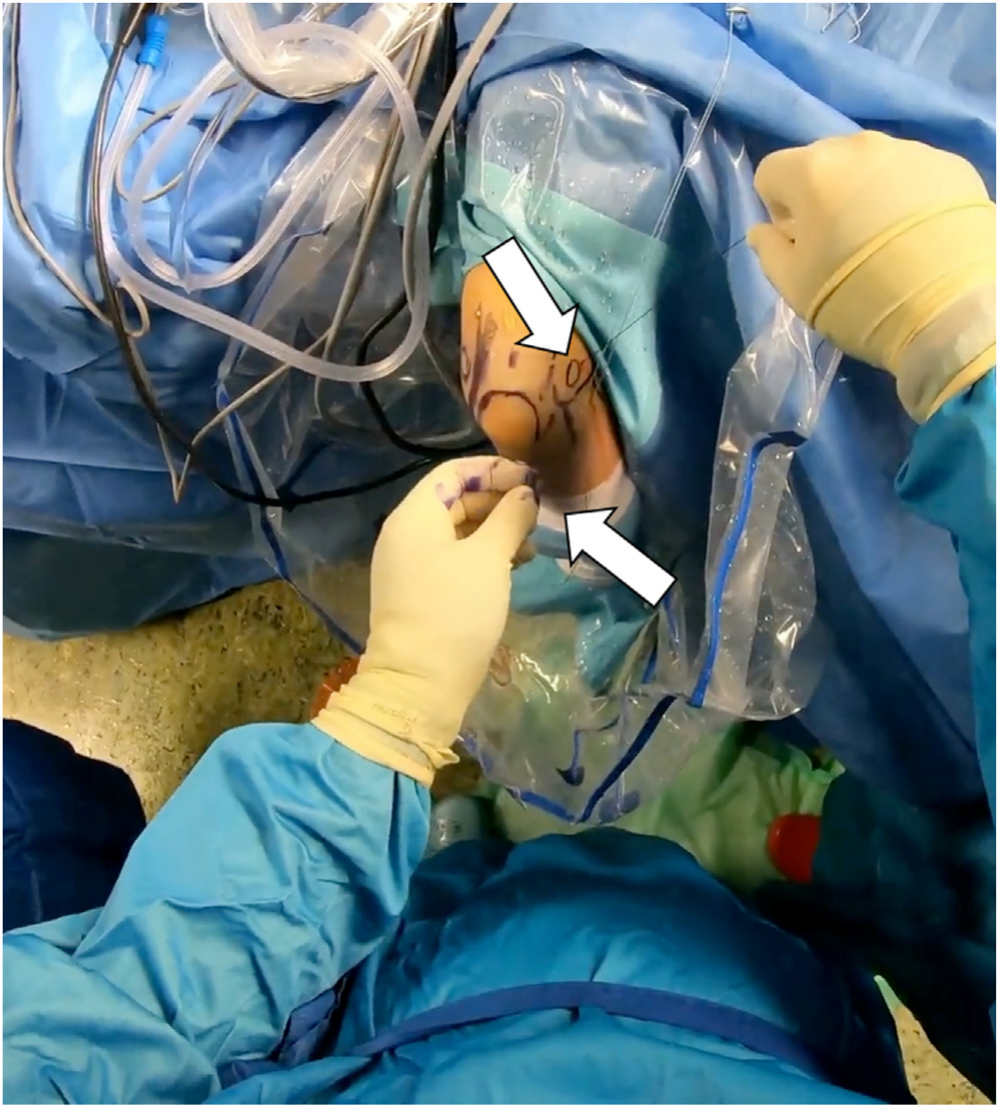
The patient is in the prone position; the right arm is positioned in an arm-holding device. The surgeon looks at the right arm from above; the arthroscopic access portals are marked. The surgeon holds the corresponding suture anchors in both hands. The patient’s arm is positioned for arthroscopic LUCL reconstruction, with sutures passed through the radial collateral ligament, common extensor origin, and LUCL using a 14-gauge needle and retrieved through an accessory portal. Horizontal mattress sutures restore tension, and additional imbrication resolves residual instability. Take-home message: Precise suture placement and tension restoration are crucial for optimal stabilization in LUCL reconstruction with augmented LCL imbrication. (LUCL, lateral ulnar collateral ligament.)

For LCL imbrication, a polydioxanone suture is shuttled using a 14-gauge needle, entering at the lateral epicondyle’s center, through the capsule and LCL complex, and retrieved via the lateral portal (Video 1). A second suture starts at the ulna’s bony edge below the LUCL insertion, passing through the anconeus, capsule, and LUCL, with both ends retrieved at the lateral portal (Fig 6).

Then, the lasso is shuttled through the entire course of the LUCL. This is replaced with a loop of apolydioxanone suture to form a single loop-suture strand that stretches from the lateral epicondyle to the distal attachment of the LUCL including the anconeus muscle. Finally, the 4 suture ends are drawn out subcutaneously through the lateral portal (Video 1).^10^

**Fig 7:**
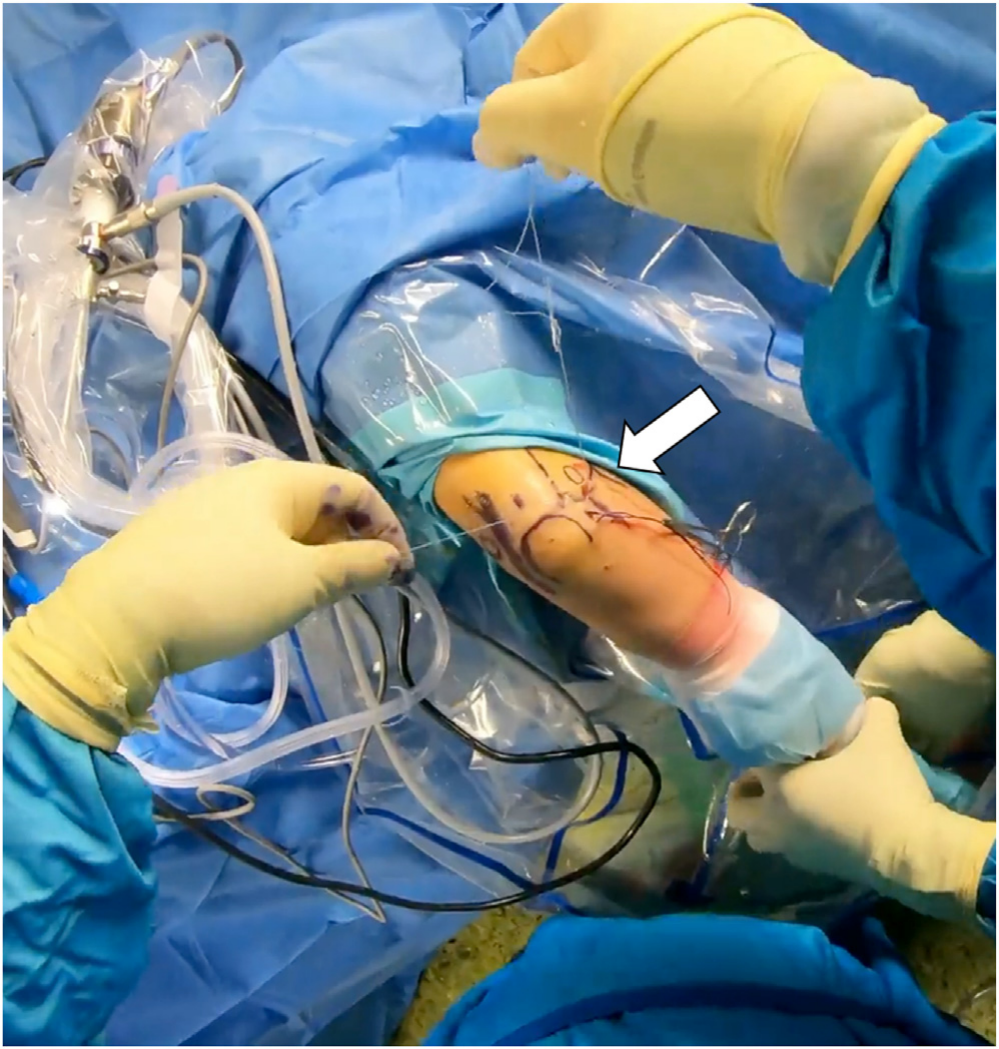
This figure shows the arm fixed in an arm rest with the elbow at 90°, highlighting anatomical landmarks and portals. Sutures are placed during arthroscopic examination to correct rotational instability through supination and varus stress. Once stability is achieved, sutures are knotted and secured, with the elbow in 30° flexion and full supination. The arthroscope is removed, knots are hidden, and skin is sutured Take-home message: Precise arthroscopic techniques, including suture placement, stability testing, and secure knotting, are essential for successful lateral collateral ligament reconstruction and optimal elbow function.

Rotational instability is then tested by supinating the forearm and applying varus stress (Fig 7). If instability is resolved with tightened sutures, they are knotted together. After verifying LCL reconstruction, the elbow is set in 30° flexion and full supination, and the sutures are secured with sliding knots. The arthroscope is removed, knots are concealed, and the skin is sutured over the portals. The patient is then fitted with a dynamic brace to support healing.

## Discussion

This treatment algorithm offers a structured approach for managing PLRI of the elbow, considering the integrity of the CEO to develop individualized surgical strategies and improve patient outcomes. The described procedure, established by Dr. Koukos, has proven effective for treating elbow instability.^10^ For better intraoperative visibility during arthroscopy, a direct posterolateral portal is recommended. Diagnostic tests, including arthroscopic rotatory instability test, pull, and trocar tests, should be performed to assess joint stability.

The minimally invasive nature of this technique preserves tissue, leading to a shorter rehabilitation phase and reducing the risk of postoperative mobility restrictions.^13^ However, it is limited to treating grade 1 and 2 PRLI cases. The procedure's steep learning curve requires the surgeon to have advanced arthroscopic skills as the result of its technical complexity. Advantages and disadvantages are listed in Table 1, and pearls and pitfalls are discussed in Table 2.

**Table 1:** Advantages and Disadvantages of the Arthroscopic LUCL Plication/Reconstruction With Augmented LCL Imbrication

Advantages	Disadvantages
Time to recovery appears shorter	Experience of surgeon is essential
Improved preservation of CEO	Flat learning curve
Comprehensive examination to obtain an overview before starting the main procedure	Complex and demanding procedure
Minimally invasive, tissue-preserving operation	Only suitable for PRLI grades I and 2
Open reduction seems to be beneficial in cases with CEO rupture and retraction	

CEO, common extensor origin; LCL, lateral collateral ligament; LUCL, lateral ulnar collateral ligament; PRLI, posterolateral rotatory instability.

**Table 2:** Pearls and Pitfalls of the Arthroscopic LUCL Plication/Reconstruction With Augmented LCL Imbrication

Pearls	Pitfalls
Individualized diagnosis and treatment	Potential nerve damage or damage of the soft tissue
Return to sports and work appears shorter	Susceptible to errors with inexperienced surgeons

LCL, lateral collateral ligament; LUCL, lateral ulnar collateral ligament.

## Disclosures

All authors (C.K., D.S., J.W., I-H.J., C.Z., K.D., D.L.) declare that they have no known competing financial interests or personal relationships that could have appeared to influence the work reported in this paper.

## Supplementary Data

Video 1 Shown is an arthroscopic lateral ulnar collateral ligament plication or reconstruction with augmented lateral collateral ligament imbrication performed by the first author. The arthroscopic view as well as the handling on the patient itself and the necessary insertion of the sutures and the corresponding knotting procedure are shown.
